# The effect of Matrigel as scaffold material for neural stem cell transplantation for treating spinal cord injury

**DOI:** 10.1038/s41598-020-59148-3

**Published:** 2020-02-13

**Authors:** Jiuling Wang, Ruiliang Chu, Na Ni, Guoxin Nan

**Affiliations:** 1 Department of Pediatric Research Institute, Children’s Hospital of Chongqing Medical University, Ministry of Education Key Laboratory of Child Development and Disorders, Chongqing, 400014 China; 2 China International Science and Technology Cooperation base of Child development and Critical Disorders;Chongqing Engineering Research Center of Stem Cell Therapy, Chongqing, China; 3 Present Address: Department of Orthopaedics Children’s Hospital of ChongqingMedical University, Chongqing, China

**Keywords:** Neural stem cells, Experimental models of disease

## Abstract

Traumatic injury to the spinal cord causes permanent loss of function and major personal, social, and economic problems. Cell-based delivery strategies is a promising approach for treating spinal cord injury (SCI). However, the inhospitable microenvironment in the injured spinal cord results in poor cell survival and uncontrolled differentiation of the transplanted stem cells. The combination of a scaffold with cells has been developed with a tendency for achieving greater survival and integration with the host tissue. We investigated the effect of Matrigel combined with neural stem cells (NSCs) *in vitro* and *in vivo*. We compared the effect of different types of scaffold on the survival and differentiation of brain-derived NSCs in an *in vitro* culture. Subsequently, NSCs were transplanted subcutaneously into nude mice to detect graft survival and differentiation *in vivo*. Finally, phosphate-buffered saline (PBS), Matrigel alone, or Matrigel seeded with NSCs was injected into 48 subacute, clinically relevant rat models of SCI (16 rats per group). Matrigel supported cell survival and differentiation efficiently *in vitro* and *in vivo*. SCI rats transplanted with NSCs in Matrigel showed improved behavioral recovery and neuronal and reactive astrocyte marker expression levels compared to PBS- or Matrigel-transplanted rats. Functional repair and neuronal and reactive astrocyte marker expression was slightly improved in the Matrigel-alone group relative to the PBS group, but not statistically significantly. These data suggest that Matrigel is a promising scaffold material for cell transplantation to the injured spinal cord.

## Introduction

Spinal cord injury (SCI) is a common disease of the central nervous system (CNS) that can result in devastating and permanent loss of neurological function due to the failure of axonal regeneration after injury, thereby interrupting the connection between the brain and the body. Paraparesis and paralysis are the most common symptoms clinically. It is widely known that SCI pathology relies not only on the primary mechanical damage, but also on certain secondary injuries, including ischemia, anoxia, excitotoxicity, inflammation, and cavity and glial scar formation^1,2^. To date, there is no effective therapy for SCI, despite much effort^3^. Eliminating further damage to the spinal cord is the current therapeutic approach for the SCI patient^4^.

Since the 1960s, when it was discovered that specific regions of the adult human brain maintain the capacity for neuroregeneration^5,6^, neural stem cells (NSCs) have been isolated from several areas in the CNS^5,7^, contributing to new treatment opportunities for regenerative processes in the injured spinal cord. Cell-based therapies can not only replace and/or repair the damaged cells, but also indirectly secrete factors that modify the environment, rendering it more conducive for regeneration^8,9^. However, the efficacy of only stem cell transplantation is limited mainly due to the inhospitable microenvironment at and around the lesion site, including inhibitory molecule upregulation, glial scar formation, inflammation, and the absence of astrocytes for guiding axon regrowth, which lead to limited cell survival, uncontrolled differentiation, and ineffective integration into the host tissue^10,11^. Even though specific factors such as growth factors improve cell survival and promote oligodendrocytic differentiation of brain-derived neural stem and progenitor cells (NSPCs), the issues of catheter patency, scarring, compression, infection, and the formation of proliferative meningeal lesions around the catheter insertion site persist^12,13^.

Biomaterials may modify the unfavorable microenvironment in the lesion site by serving as delivery vehicles for cells and/or biomolecules. At the same time, biomaterials can also provide structural support, which may be helpful for filling the gap between the cut ends of the ruptured spinal cord^14,15^. Numerous studies on solid scaffolds that can be artificially modified have shown that the viability of cells in the scaffold material were highly improved, and the scaffold material can also guide the new axons to extend in the proper direction. However, in the models of impact/compression SCI, which are relevant clinically, an injectable biomaterial containing cells would be more advantageous^16–18^. Matrigel is a solubilized basement membrane protein that begins to form a three-dimensional (3D) gel above 10 °C. It is mainly composed of laminin, followed by collagen IV, heparan sulfate proteoglycans, entactin/nidogen, and abundant growth factors. Fragments of laminin-1, collagen IV, and other matrix proteins can not only promote the differentiation of many cell types^19–21^, but can also affect the outgrowth of differentiated cells from tissue explants^22^. In a transected adult rat spinal cord, a Schwann cell–seeded Matrigel scaffold not only promoted axon growth in the vicinity of the grafts but also from neurons rostral to the explant^23^. Interestingly, when Matrigel was used to deliver human neural precursor cells to treat a focal cerebral ischemia model, the authors reported reduced cavity size, greater cell survival, primarily neuronal differentiation of transplanted cells, and improved behavioral outcome^24^. To determine whether Matrigel can serve as an ideal scaffold material for cell transplantation in SCI, we first examined cell survival in different scaffold materials: Matrigel, absorbable collagen sponge, POC/TCP (tricalcium phosphate/poly [1, 8 octanediol-co-citrate)]), and *in vitro* co-culture of primary NSCs. Then, we inoculated nude mice subcutaneously with primary NSCs combined with Matrigel to detect cell survival and differentiation in Matrigel; we evaluated functional recovery and tissue response after using Matrigel to deliver brain-derived primary NSCs to treat a rat model of compression SCI *in vivo*.

## Materials and Methods

### Primary neural stem/progenitor cell culture

Primary NSCs were isolated from the hippocampus of newborn Sprague-Dawley (SD) rats within 24 hours. The hippocampal tissue was mechanically dissociated and seeded in free-floating culture in Dulbecco’s modified Eagle’s medium (DMEM)/F12 (Gibco) supplemented with 2% B27 (Gibco-Invitrogen), 1% penicillin/streptomycin (Gibco-Invitrogen), 20 ng/ml epidermal growth factor (EGF, Sigma-Aldrich), and 20 ng/ml fibroblast growth factor-2 (FGF-2, Sigma-Aldrich). Half of the medium was changed every 2 days, and the neurospheres generated were passaged at a ratio of 1:2 every 4–6 days via mechanical dissociation in serum-free medium.

### *In vitro* characterization of primary NSCs

Passage 3 neurospheres were plated in 24-well plates containing glass coverslips pre-coated with poly-L-lysine (Sigma-Aldrich), incubated at 37 °C in a 5% CO_2_ incubator, and allowed to attach for 5–6 hours. Then, a portion of the neurospheres was used for fluorescence immunocytochemistry; the other was seeded in EGF- and FGF-free culture medium containing 10% fetal bovine serum (FBS), and cultured for 2 weeks. Immunocytochemistry experiments were performed as previously described^25^. The cells were washed with phosphate-buffered saline (PBS) and fixed with 4% paraformaldehyde (PFA) in PBS for 15 minutes, and then washed in PBS and incubated in blocking solution (PBS containing 10% FBS and 0.1% Triton X-100). Subsequently, the cells were incubated overnight at 4 °C with the following primary antibodies: Mouse anti-nestin monoclonal antibody (1:400; Abcam) was used to identify the NSCs. Rabbit anti-GFAP (glial fibrillary acidic protein) monoclonal antibody (1:1000; Abcam) was used to detect the astrocytes; Rabbit anti-MAP2 monoclonal antibody (1:300; Abcam) was used to detect the neurons. After three washes in PBS, the cells were incubated for 1 hour with FITC (fluorescein isothiocyanate)-conjugated goat anti-rabbit and TRITC (tetramethylrhodamine isothiocyanate)-conjugated goat anti-mouse secondary antibodies at room temperature. After three washes with PBS, the cells were coverslipped with Vectashield mounting medium containing 4’, 6-diamidino-2-phenylindole (DAPI, Sigma-Aldrich) nuclear counterstain. A Nikon Eclipse TE300 microscope and a Zeiss LSM 510 confocal microscope were used to examine the immunofluorescent staining.

### Cell culture in Matrigel1

Passage 3 neurospheres were collected and mechanical force was used to form the neurospheres into single-cell suspensions, which were then inoculated in a Petri dish. When cell proliferation was 50%, an appropriate amount of Ad-GFP (green fluorescent protein) virus was added. GFP expression in the cells was observed under a fluorescence microscope at 1, 3, and 5 days after infection. If GFP expression was inconsistent, more Ad-GFP virus was added. The virus infection rate was about 80% on day 5 after infection. At this time, the cells were collected and inoculated with Matrigel, TCP/POC, or absorbable sponge at a density of 5 × 10^4^ cells per ml in a 24-well culture plate (1 ml/well). The cells were incubated at 37 °C in 5% CO_2_. Cell survival was observed under fluorescence microscopy at 1, 3, and 14 days after inoculation.

### Subcutaneous NSC tumor in nude mice

Passage 3 neurospheres were mechanically separated, counted using a cell counter, and gently resuspended in a 1:1 (v:v) solution of PBS:Matrigel at a final density of 5 × 10^6^ cells per ml. The resuspended cell suspension was inoculated subcutaneously into nude mice (0.2 ml/site). The inoculation site grew in unequal masses, and the mice were sacrificed to remove the mass after 2 weeks of feeding in specific pathogen–free conditions SPF. The tumors were stored in 4% PFA at 4 °C. Routinely dehydrated and paraffin-embedded sections were stained using hematoxylin–eosin (H&E) and immunofluorescence, and rabbit anti–β-tubulin III was used to detect neurons.

### Animals and SCI injury model

Forty-eight adult female wild-type SD rats (weight: 200–220 g) provided by the Animal Center of Chongqing Medical University were used in the present study. This study was carried out in strict accordance with the National Institutes of Health Guide for the Care and Use of Laboratory Animals. All animal procedures were approved by the Institutional Animal Care and Use Committee accredited by the Association for the Assessment and Accreditation of Laboratory Animal Care International in China and the Experimental Animal Committee of Chongqing Medical University (Permit numbers: SCXK [Yu] 2012–0001 and SYXK [Yu] 2012 – 0001). All animals were bred at room temperature (between 20 °C and 25 °C) with 40–60% relative humidity and a 12–12-hour day–night cycle; water and food were offered *ad libitum*. The rats were anesthetized intraperitoneally using 10 g/l sodium pentobarbital (40 mg/kg) and were secured on an operating table before skin preparation and routine sterilization. The T10 vertebral level spinal cord was exposed by laminectomy, and a modified Allen method was used to induce contusion injury as described previously^26^. Briefly, the exposed spinal cord was covered with a thin copper slice vertical to a graduated glass tube. A 10-g metal block was dropped onto the copper slice from a height of 2.5 cm, and the copper slice was removed immediately. If the spinal cord was congested and the rat’s tail curved or swung after the impact, injury induction was considered successful. During the surgery, the rats were kept on a 37 °C heating pad to maintain body temperature. To prevent postoperative infection, all rats were injected intraperitoneally with penicillin (20,000 U/kg) twice daily during the first 7 days after surgery. Manual bladder expression was carried out at least three times per day until voluntary urination was recovered.

### Transplantation

All rats underwent a second operative procedure 1 week post-SCI. The injured animals were randomly divided into three groups based on their Basso-Beattie-Bresnahan (BBB) open field locomotor score (see below, functional analysis) to ensure equivalent deficits across groups before treatment. The three groups were: PBS control (n = 16), Matrigel alone (n = 16), or Matrigel with primary NSCs (n = 16, NSCs/Matrigel). All rats were anesthetized as described above, and cell suspensions were pre-mixed with Matrigel, resulting in a final cell concentration of 5 × 10^4^ cells/µl in a 50:50 (v:v) solution of PBS:Matrigel. After the previous operative site was re-exposed, PBS (10 µl), Matrigel (10 µl), or NSCs/Matrigel (5 × 10^5^ cells suspended in 10 µl Matrigel) was transplanted into the center of the lesion site using a 10-µl Hamilton syringe. Then, the lesion was closed routinely. Eight rats per group were assessed weekly using the BBB scale from week 1 to 9 post-transplantation, and the remaining rats (eight per group) were sacrificed for histological analysis at 4 weeks post-transplantation.

### Behavioral analysis

Two individuals blinded to the experimental conditions performed behavioral analysis using the BBB scale^27,28^. The BBB scale ranges 0–21 points; a score of 0 indicates no hindlimb movement; a score of 21 indicates normal gait. The test was performed pre-injury and pre-transplantation, and then weekly for 8 weeks post-transplantation. The mean and standard error (SE) of the BBB scores were calculated for every group at every time point, and Student’s *t*-test (one-tailed) was used to assess significant differences among the three groups.

### Histology and immunohistochemistry

Rats from each group were sacrificed at 1, 2, or 4 weeks post-transplantation for immunohistochemical analysis. The rats were injected subcutaneously with sodium pentobarbital, and then transcardially perfused with 0.9% saline solution and then 4% PFA in PBS (pH 7.4). Next, a 1.5-cm segment of spinal cord tissue, including the T10 transection site, was removed and immersed in 4% PFA for additional fixation for at least 24 hours. Samples were dehydrated in a gradient ethanol series, embedded in paraffin, and sectioned sagittally (n = 5 per group) or transversely (n = 3 per group) into 4 µm thick sections.

The sections were stained with H&E for general morphology studies. Immunohistochemistry was performed as described previously^29,30^. The sections were deparaffinized in xylene and rehydrated in an ethanol gradient, and then subjected to heat-induced antigen retrieval. To inactivate endogenous peroxidases, the sections were exposed to 0.3% H_2_O_2_ for 30 minutes at room temperature. After three washes in PBS, the sections were pre-incubated with 5% normal goat serum for 1 hour to decrease non-specific antibody binding. Next, the sections were stained with primary antibody overnight at 4 °C as follows: mouse monoclonal anti-nestin antibody (1:5000; Abcam) for NSCs, rabbit monoclonal anti-GFAP (1:2000, Abcam) for astrocytes; pan-neuronal marker including rabbit anti-NeuN monoclonal antibody (1:3000; Abcam), rabbit anti-MAP2 monoclonal anibody (1:600, Abcam), and mouse anti–neurofilament 200 (NF-200, 1:400, Abcam). The primary antibodies constituted a neuronal marker specific to axons (neurites), dendrites, and the neuronal cell body. The next day, the sections were washed using PBS and incubated with biotinylated anti-mouse/rat secondary antibody (1:1000, Zhongshanjinqiao). 3,3′-Diaminobenzidine (DAB, Zhongshanjinqiao) was used for the staining visualization, followed by incubation with secondary antibody. For the immunofluorescence studies, the sections were incubated with Alexa 488–conjugated secondary antibody after incubation with the primary anti–NF-200 antibody above and PBS washing. DAPI (1:200, Sigma-Aldrich) was used for nuclear staining.

### Statistical analysis

*In vitro* cell viability data are presented as the mean ± standard deviation; functional data are presented as the mean ± SE of the mean (SEM). SPSS version 17.0 (SPSS Inc, Chicago, IL, USA) was used for statistical analysis. Two-way repeated-measures analysis of variance (ANOVA) comparing groups versus time points followed by post-hoc pairwise multiple comparisons using the Bonferroni method was used to analyze the functional tests; one-way ANOVA followed by pairwise multiple comparisons using the Bonferroni test was used to analyze cell survival in the scaffolds. P < 0.05 was considered significantly different.

## Results

### *In vitro* culture and differentiation of NSCs

**Figure 1:**
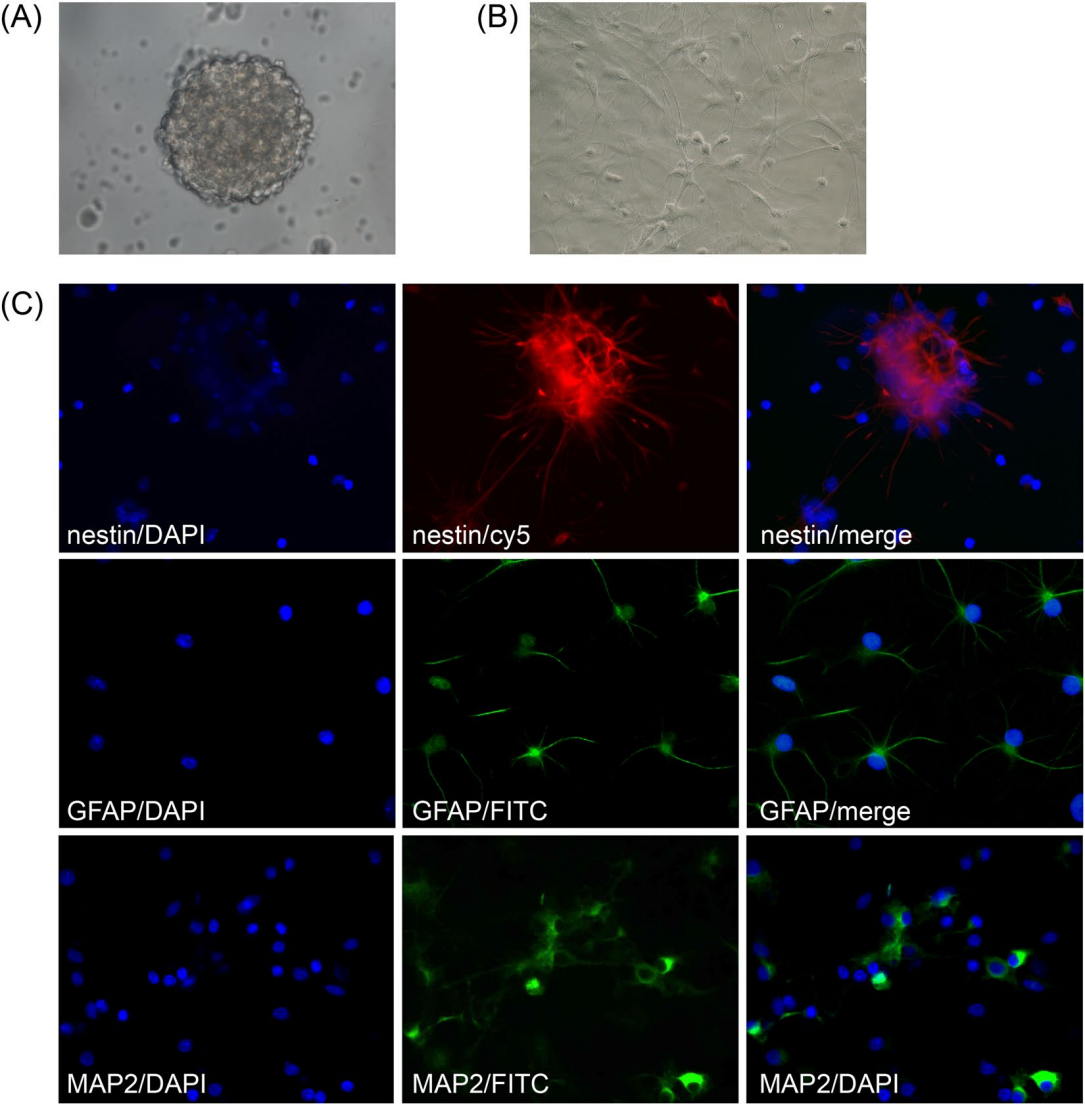
(**A**) Morphology of neurospheres under optical microscopy after 7-day culture (×400). (**B**) Differentiation of primary NSCs. Cells were cultured in serum-containing medium for 7 days (×200). (**C**) Immunofluorescence of primary NSCs. Cells were immunostained for nestin (red, ×400), GFAP (green, ×400), and MAP2 (green, ×400). Nuclei were stained with DAPI (blue).

Here, NSCs derived from the hippocampus of newborn SD rats were transplanted with Matrigel into injured spinal cords to evaluate the possibility of Matrigel serving as cell scaffold material. When the cells had been cultured for 7 days, neurosphere generation consisting of hundreds of cells was observed (Fig. 1A). Immunocytochemistry showed high nestin expression in the neurospheres (Fig. 1C). When the neurospheres were cultured in medium containing 10% FBS for 2 weeks, cells emigrated from the neurospheres, resulting in loss of neurosphere structure. These cells attached to the wall and differentiated into varieties of cells, forming a 3D network structure (Fig. 1B). The *in vitro* differentiation assay revealed that the NSCs could differentiate into GFAP-positive astrocytes and MAP2-positive neurons (Fig. 1C).

### Cell survival and differentiation in Matrigel

**Figure 2:**
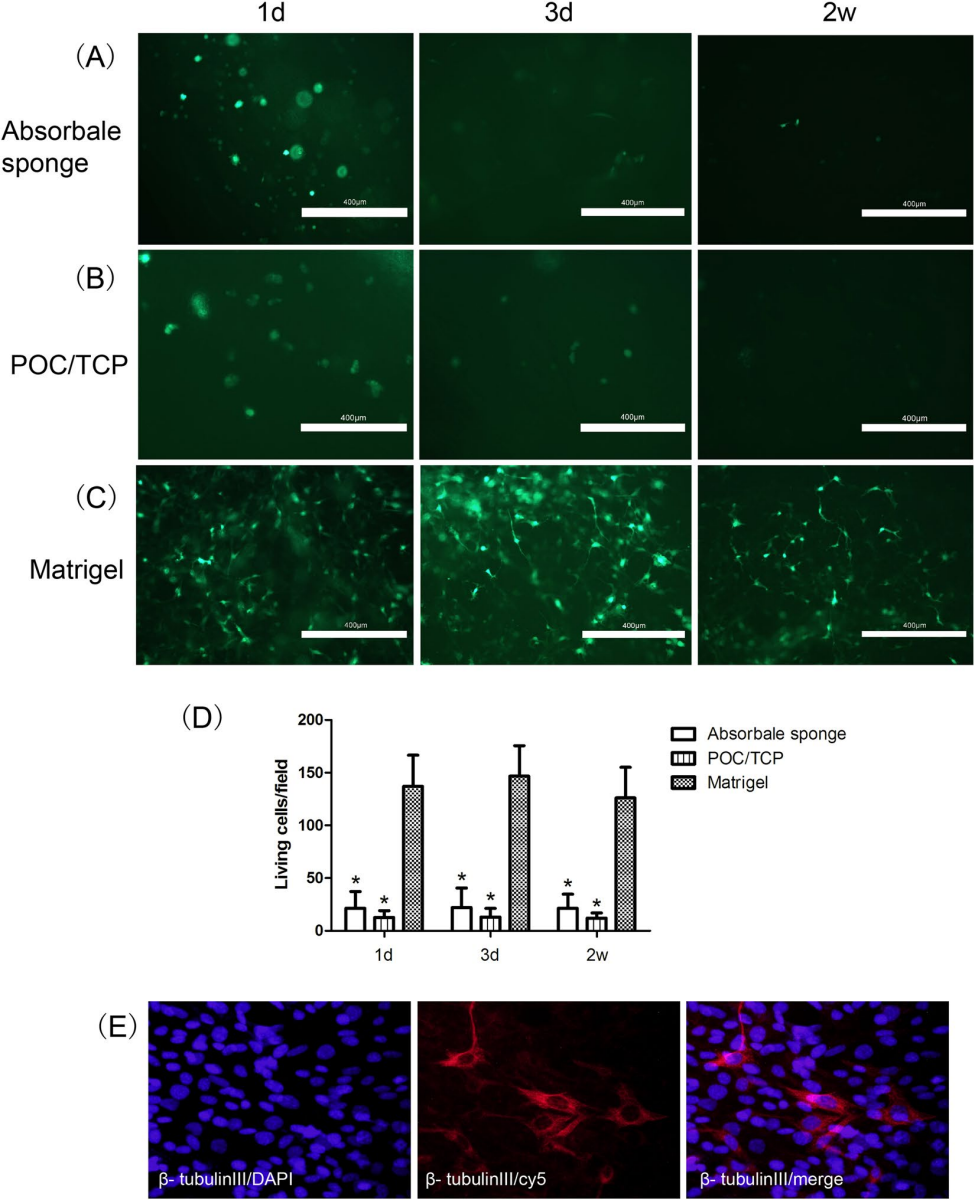
Matrigel improved cell survival compared with the absorbable collagen sponge and POC/TCP. Cells were cultured in (**A**) absorbable sponge, (**B**) POC/TCP, or (**C**) Matrigel. (**A,B**) Cell survival was poor when cultured for 3 days. (**C**) Cell survival was high after 2 weeks. Scale bars = 400 µm. (**D**) Quantification of cell survival in the different materials. Cell survival in Matrigel was significantly higher than that in the other two materials (data are the mean ± SEM; *p < 0.05). (**E**) Laser confocal microscopy images showing that primary NSCs could differentiate into neurons in Matrigel (×400).

The NSCs were cultured in Matrigel, POC/TCP, or an equal volume of absorbable sponge for 2 weeks to compare the effects of culture conditions. Vital cells were observed under fluorescence microscopy and expressed as the percentage of living cells at 1, 3, 14 days (Fig. 2A–D). The percentage of live cells in Matrigel was significantly higher than that in POC/TCP or absorbable sponge; there were no significant differences in the latter two groups at all time points. There were few live cells in POC/TCP (3.00 ± 1.00 vital cells/field) and absorbable sponge (5.00 ± 2.00 vital cells/field) by day 14, while many cells (78.00 ± 11.00 vital cells/field) still grew well in Matrigel by day 14. For *in vitro* differentiation into neuron detection in Matrigel, cells cultured in Matrigel for 7 days were immunocytochemically stained for β-tubulin III. Interestingly, β-tubulin III–positive neurons were observed in the Matrigel (Fig. 2E).

### Subcutaneous NSC tumor in nude mice

**Figure 3:**
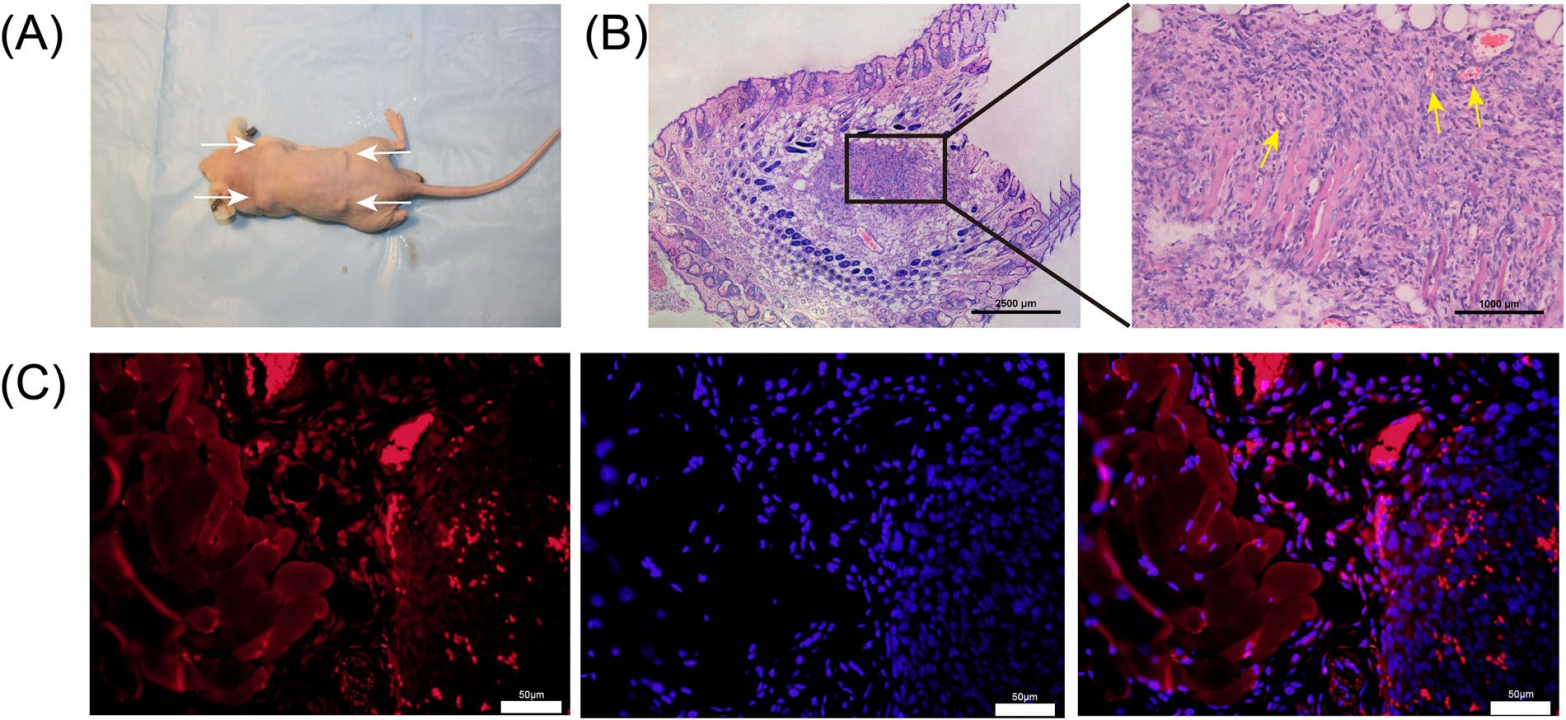
*In vivo* cell survival and differentiation of primary NSCs into neural-like cells. (**A**) NSCs/Matrigel complex was injected into nude mice (white arrows indicate the nodule location). (**B**) H&E-stained tumor sections from the transplanted NSCs showing good cell survival at 2 weeks post-transplantation (n = 5) (yellow arrows indicate new vessels). (**C**) Nodule sections were immunostained for β-tubulin III (red); nuclei were stained with DAPI (blue). Scale bars = ×200.

The NSCs/Matrigel composite was inoculated subcutaneously into the backs of the extremities of the nude mice. After 1 week, soybean-sized nodules were observed (white arrows indicate the nodule location)(Fig. 3A). The nodules were dissected and isolated for histological analysis. H&E staining showed abundant nerve cells that grew well in Matrigel, and a small number of blood vessels (yellow arrows indicate new vessels) (Fig. 3B). The nodule sections were immunostained for β-tubulin III, and some β-tubulin III–positive neuron-like cells were observed (Fig. 3C).

### BBB scores

**Figure 4:**
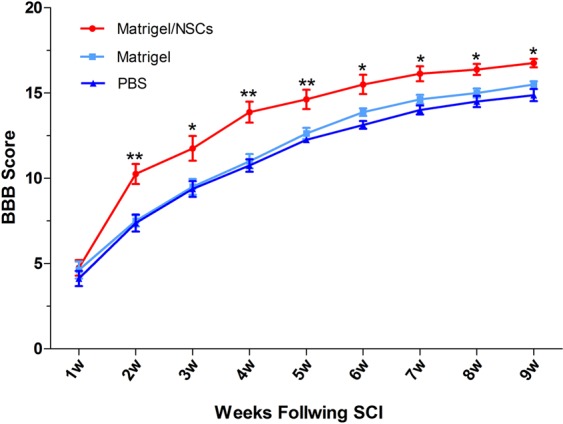
Functional recovery evaluation using BBB scores. The Matrigel/NSC group had significantly higher BBB scores compared with the other groups. The Matrigel-alone group had slightly improved BBB scores compared to the PBS group, but it was not statistically significant. (Data are the mean ± SEM; *p < 0.05; **p < 0.01.).

We used BBB scores for weekly assessment of functional recovery following SCI. The average BBB score did not exceed 6.03 (Fig. 4 and Table 1) in the three groups during the first week after SCI. Subsequently, motor function was gradually recovered at a faster rate but slowed at 4–8 weeks post-SCI. During this period, the NSCs/Matrigel group had statistically significantly improved BBB scores compared to the other two groups. It is worth noting that the Matrigel-alone group showed only slightly improved BBB scores relative to the PBS group, although this was not statistically significant. The final BBB score at 9 weeks post-SCI was 16.75 ± 0.71 in the NSCs/Matrigel group, 15.50 ± 0.53 in the Matrigel group, and 14.88 ± 0.99 in the PBS group. Therefore, rats transplanted with explants containing Matrigel showed better functional recovery.

**Table 1:** BBB Score ($\bar{x}$ ± SEM).

groups	n	1w	2w	3w	4w	5w	6w	7w	8w	9w
Matrigel with NSCs	8	4.75 ± 1.28	10.25 ± 1.67	11.75 ± 2.05	13.88 ± 1.73	14.63 ± 1.60	15.50 ± 1.61	16.13 ± 1.25	16.38 ± 0.92	16.75 ± 0.71
Matrigel alone	8	4.62 ± 1.41	7.50 ± 0.93	9.50 ± 1.31	11.00 ± 1.20	12.63 ± 0.92	13.88 ± 0.64	14.63 ± 0.74	15.00 ± 0.76	15.50 ± 0.53
PBS	8	4.13 ± 1.25	7.38 ± 1.41	9.38 ± 1..30	10.75 ± 1.04	12.25 ± 0.46	13.13 ± 0.64	14.00 ± 0.76	14.50 ± 0.93	14.88 ± 0.99

*P-*value based on one-way ANOVA followed by pairwise multiple comparisons using Student-Newman-Keuls testing.

### Cell survival in injured spinal cord

**Figure 5:**
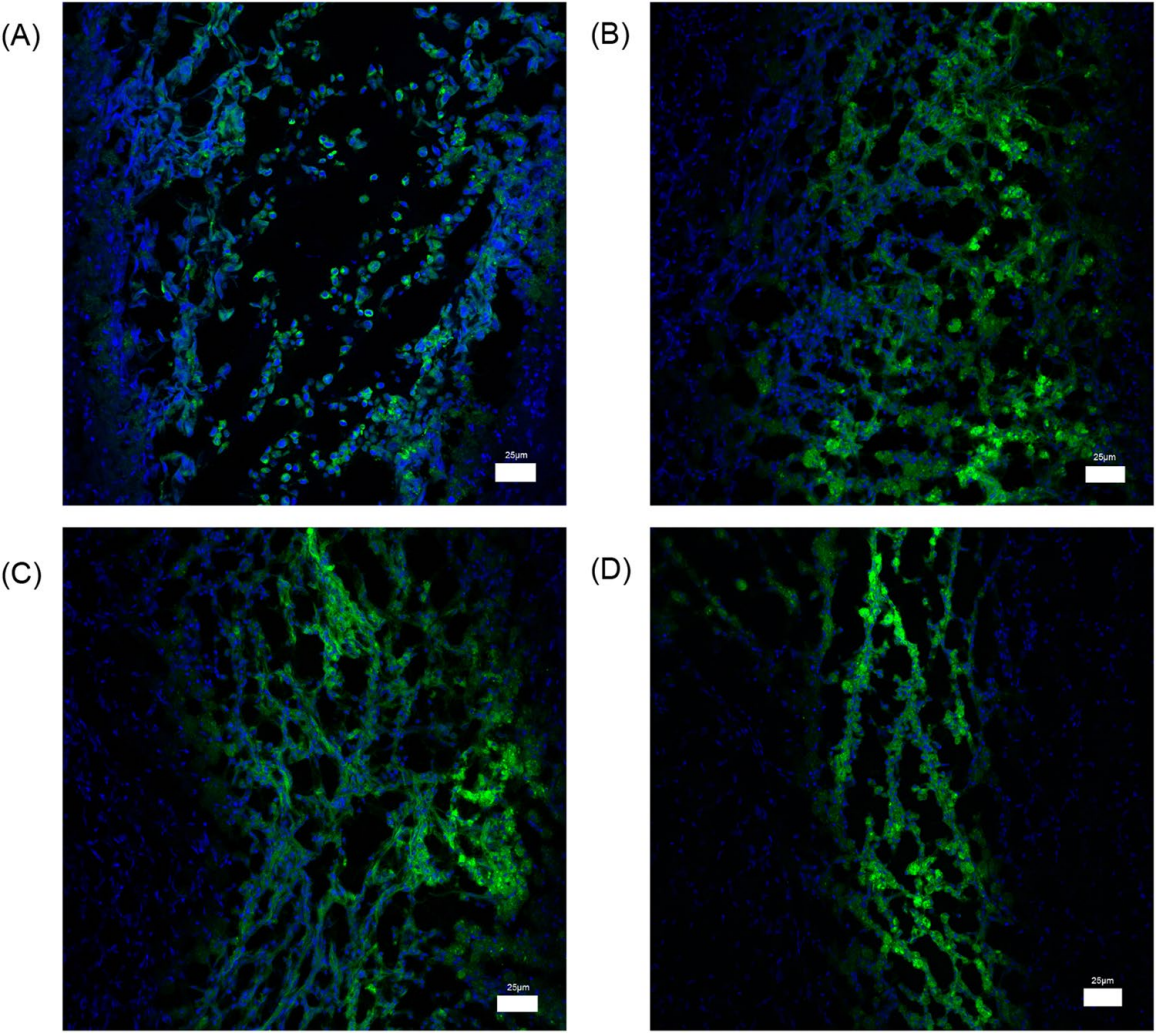
Cells were labeled with PKH67 to detect cell survival in injured spinal cords. (**A**) 1 day after transplantation. (**B**) 7 days after transplantation. (**C**) 14 days after transplantation. (**D**) 28 days after transplantation. Scale bars = ×100.

After PKH67 fluorescent labeling, the cells were observed using confocal fluorescence microscopy at 488 nm. The NSC cell membranes emitted green fluorescence. The injured spinal cord was observed using confocal fluorescence microscopy. We observed many PKH67-positive cells, appearing singly, and were round, in the cavity of the injured spinal cord 1 day after implantation (Fig. 5A). At 1 week after implantation, many PKH67-positive cells were observed and appeared elongated, irregularly shaped, fused together(Fig. 5B). At 2 weeks after implantation, a few PKH67-positive cells were observed(Fig. 5C). At 4 weeks after implantation, the number of PKH67-positive cells decreased, but the viable cells were in good condition (Fig. 5D).

### Histopathologic analysis of the spinal cord

**Figure 6:**
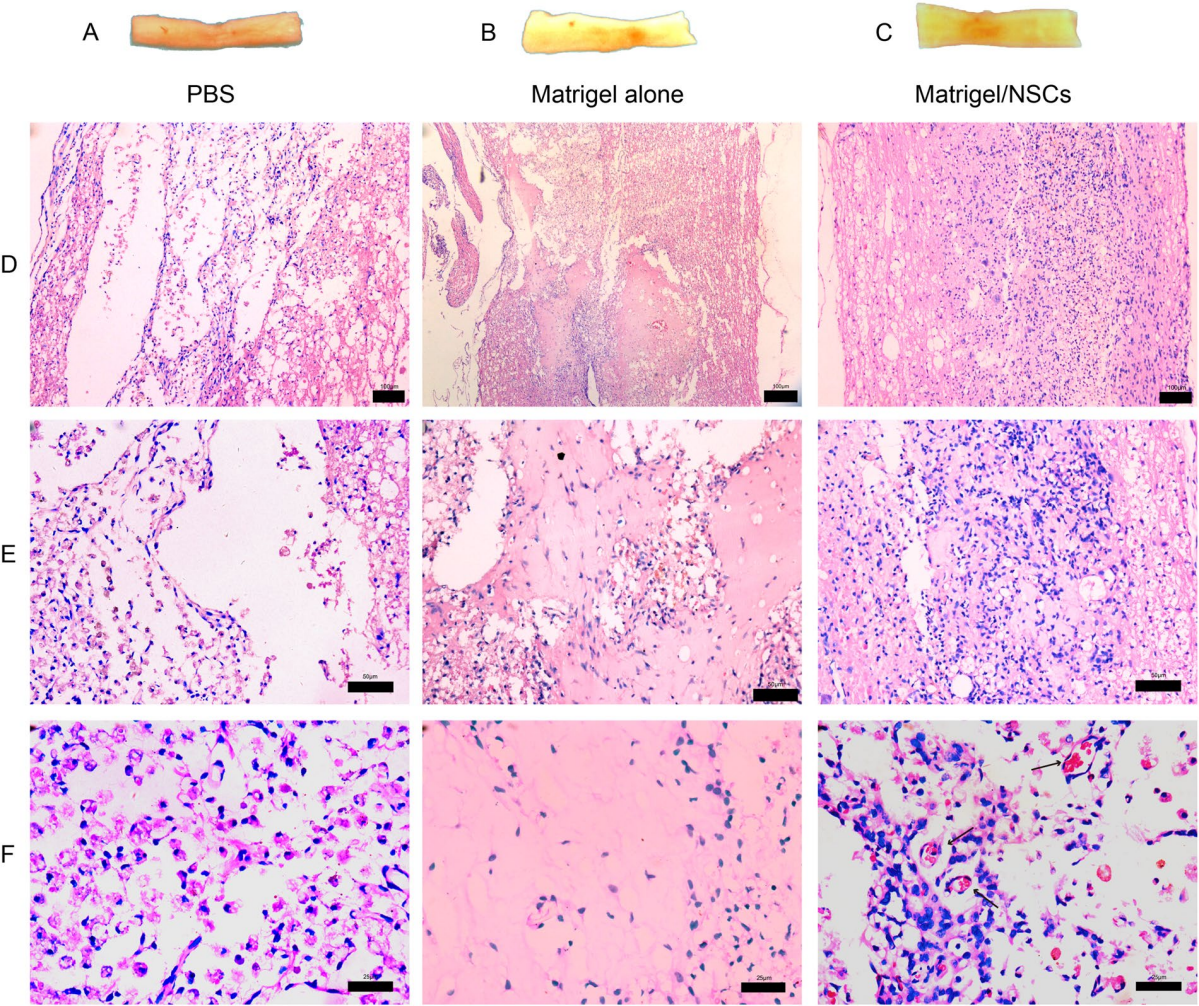
Gross morphology and H&E staining at 4 weeks post-transplant. (**A–C**) The injured spinal cord of the PBS group (**A**), Matrigel group (**B**), and Matrigel/NSCs group (**C**). (**D–F**) H&E-stained spinal cord sections at 4 weeks (×100 [**D**], ×200 [**E**], ×400 [**F**]).

There was cavity formation in the injured spinal cord of the PBS group, and the surface of the injured spinal cord was collapsed (Fig. 6A). The injured spinal cord surface of the Matrigel-alone group and NSCs/Matrigel group was plump, smooth, and not significantly collapsed (Fig. 6B,C). At 4 weeks after transplantation, the injured spinal cord sections were observed under fluorescence microscopy. In the PBS group, numerous inflammatory cells and fibroblasts had infiltrated the SCI site and cavities had formed. The Matrigel-only group had less inflammatory cell infiltration than the PBS group, and reduced tissue necrosis area. In the Matrigel-only group, the syringomyelia was filled with Matrigel and the boundary of the surrounding tissue was clear, and more cell growth could be seen in the Matrigel. The SCI site of the NSCs/Matrigel group was filled with Matrigel, and the surrounding liquefaction area was smaller than that of the other two groups. Many nerve cells had grown in the Matrigel, and the density was higher than that of the other two groups, and new blood vessels were visible (Fig. 6D–F).

### Evaluation of neuronal regeneration

**Figure 7:**
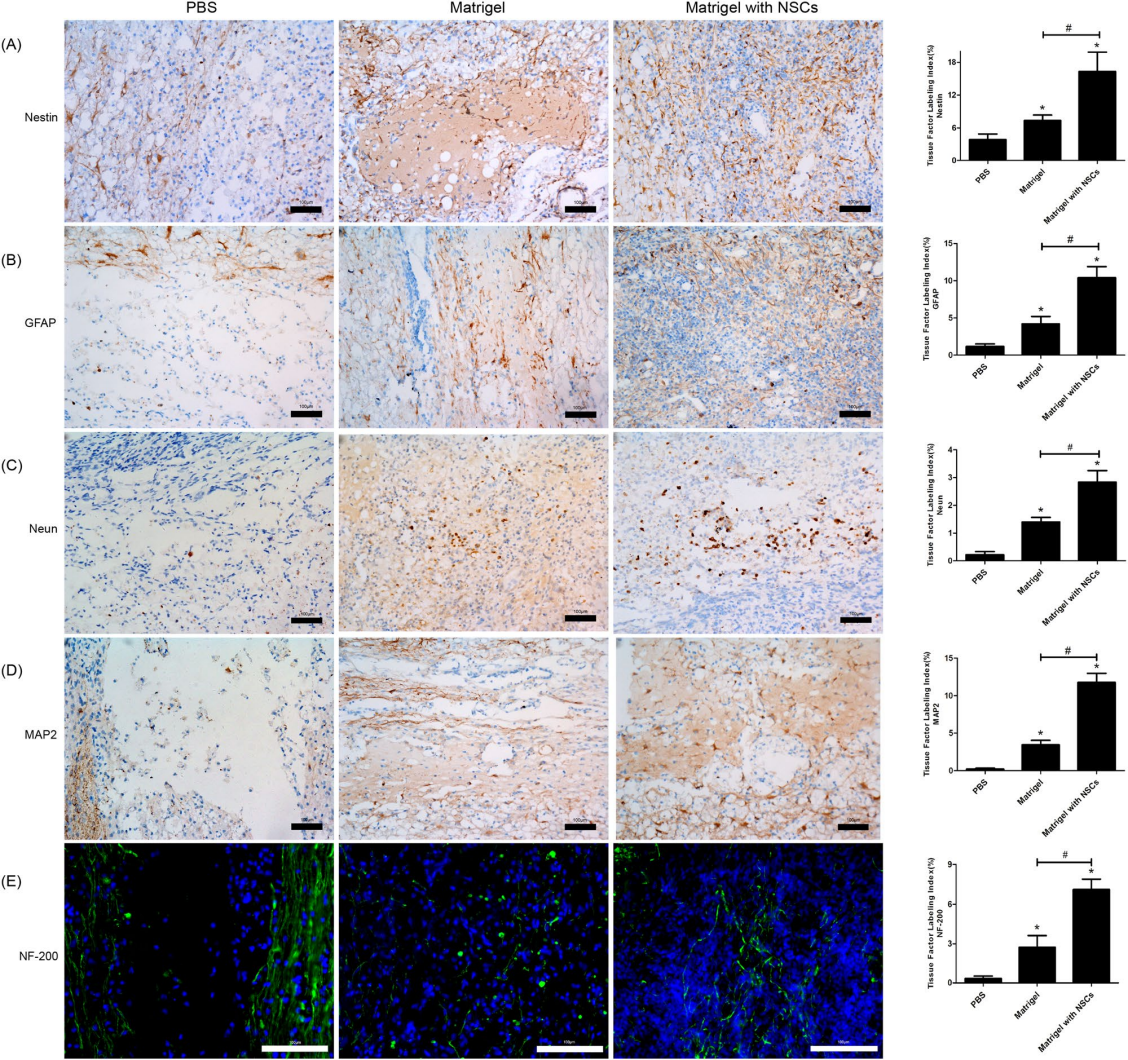
Immunohistochemistry at 4 weeks post-transplant. (**A–E**) Photomicrographs of injured spinal cord immunostained for nestin (**A**), GFAP (**B**), NeuN (**C**), MAP2 (**D**), and NF-200 (**E**) at 4 weeks post-transplant. Positive staining is visible as brown or green dots. Original magnification = ×200. (*P < 0.05 compared with the PBS group. ^#^p < 0.05).

To detect neuronal regeneration in the lesion site, we examined the nestin, GFAP, NeuN, MAP2, and NF-200 expression levels (Fig. 7). Nestin expression in the epicenter of the injured region was significantly increased in the NSCs/Matrigel group compared to that of the other groups. On the other hand, the PBS group had almost no nestin expression in the lesion epicenter, while there was a significant maximum increase in the host tissue adjacent to the lesion site. Some nestin expression was also observed in the grafts in the injured region, suggesting that endogenous NSCs had migrated into the grafts (Fig. 7A). The expression of GFAP, a marker of reactive astrocytes, was highest in the NSCs/Matrigel group and second highest in the Matrigel-alone group (Fig. 7B). The neuronal markers NeuN, MAP2, and NF-200 were almost undetectable in the PBS group; however, they were significantly increased in the NSCs/Matrigel and Matrigel-alone groups, although the increase in the former was more marked (Fig. 7C–E). The expression of NF-200, a marker of nerve fibers, showed that nerve fibers could grow and extend in the grafts. Altogether, more nerve cell markers were detected in the NSCs/Matrigel group than in the other groups.

## Discussion

After SCI, there is hemorrhage, loss of nerve cells, cavity formation, and scarring in the injured spinal cord. Scar formation prevents the growth and passage of nerve fibers. Cell therapy is an emerging technology for replacing and/or regenerating injured tissues in the CNS. Nevertheless, the low survival rate of grafted cells resulting from mechanical damage, acute inflammation, and immunological rejection is one a major obstacle to the success of that therapeutic approach^10,11^. The key to cell survival is the scaffold material in the transplant. The scaffold material used in spinal regeneration in recent years is commonly a poly(D,L-lactic-co-glycolic acid)^31^ poly-beta-hydroxybutyrate^32^ electrospun construct based on self-assembling peptides^33^. However, these materials are solid and could cause secondary damage to the injured spine during transplant surgery. Matrigel, first evaluated *in vitro* for 3D cell culture and as a 3D cell culture scaffold, is liquid at low temperatures (<4 °C) and becomes solid at a biological temperature (>10 °C). Therefore, the liquid Matrigel can be mixed with NSCs and injected into the hollowed-out portion of the spinal cord, and will not cause secondary damage to the injured spine. As the temperature increases, the Matrigel gradually solidifies, and the NSCs are evenly distributed. We are particularly interested in whether cells can survive for a long time and differentiate into neurons after transplantation.

Matrigel, a natural extracellular matrix, is biocompatible and biodegradable. In the initial *in vitro* experiment, we compared the effects of Matrigel, POC/TCP, and absorbable sponge in an *in vitro* cell culture. We found that NSCs could survive for a long time and differentiated into neural-like cells in Matrigel, suggesting that Matrigel can support cell survival and differentiation *in vitro*. Subsequently, good cell growth and survival was observed when we transplanted NSCs into nude mice or injured spinal cords, which showed similar effects with the *in vitro* experiments. Uemura *et al*. compared the supportive effects of different matrices *in vitro*: collagen IV, poly-L-ornithine/laminin, growth factor–reduced Matrigel (grftMG), and PuraMatrix, and found that grftMG supported embryonic stem cell–derived neural precursor cells more efficiently than the other matrices^34^. Moreover, Cao *et al*. confirmed that Matrigel alone could support cell survival for up to 5 months^34^. Our results are consistent with these studies. The mechanisms of this supportive effect of Matrigel may include: rescue of dying cells, promotion of cell proliferation, and blockade of invading inflammatory or immunological cells. Of course, the growth factors present in Matrigel may also contribute to the supportive effects.

Cystic cavity resulting from maceration and necrosis of the injured spinal cord was clearly filled with Matrigel as well as a number of cells, and the cavity was reduced in the Matrigel-alone group or NSCs/Matrigel group (Fig. 6). It suggests that Matrigel is advantageous in that it can resemble the spinal cord tissues in terms of mechanical properties, be easily implanted by simple injection, and its shape conforms well with the surrounding tissues. To date, a variety of scaffolds, including solid scaffolds, have been used for SCI repair. Solid scaffolds are more widely used in complete transection or hemisection models^16–18^. However, a rat impact model, which is more relevant clinically, showed that it is difficult to ensure that solid scaffold material is completely consistent with the injured parts, and that it is easy to cause secondary damage to the surrounding tissue. Accordingly, injectable scaffolds such as Matrigel are more advantageous.

Here, immunohistochemical assessment of the injured spinal cords showed that expression of the neural cell markers nestin, GFAP, NeuN, MAP2, and NF-200 was highest in the NSCs/Matrigel group, followed by that in the Matrigel-alone group. Nestin expression mainly increased in spared tissues adjacent to the epicenter of the damaged site in the PBS group, while more nestin expression was observed in the graft site in the Matrigel-alone group. This may be explained by Matrigel blocking the invading inflammatory or immunological cells and providing the best environment for the NSCs. In addition, Matrigel itself contains abundant growth factors, such as FGF-2, EGF, and transforming growth factor-beta (TGF-β), which promote NSC proliferation^34^. As expected, nestin expression in the NSCs/Matrigel group was greatly increased compared to that of the other groups, which contrasted with the findings of Park *et al*.^35^, who showed that nestin expression increased only in the Matrigel-alone group. A different cell source, followed by differentiation efficiency of the transplanted cells to neuronal cells or other specific cells may be a possible explanation for this difference. A longer experimental time course may have resulted in lower nestin expression as the NSCs differentiated into other cell types in the NSCs/Matrigel group. Previous studies^36,37^ have shown higher GFAP intensity as compared to normal spinal cord at 3–11 weeks after transplantation. However, in our study, and consistent with Kaneko *et al*.^30^, GFAP expression was extremely low in the injury site at 9 weeks post-injury in the PBS group, while it was greatly increased in the NSCs/Matrigel group (Fig. 7). Recent studies have stated that increased GFAP expression does not only affect astrogliosis, but also other factors, including inflammatory cells and other glial cells that may interact with reactive astrocytes works. Reactive astrocytes are not a sufficient condition, but a necessary one, for astrogliosis^35^. Astrocytes facilitate synapse formation and neuron physiological maturation and promote axonal regeneration^38,39^. In the Matrigel-alone group, there was an increase in other markers not only for reactive astrocytes but also for neuronal markers. As shown in Fig. 7, NSCs/Matrigel promoted increased neurons, while little neuronal regeneration was observed in the PBS group. High neuron loss is usually encountered after SCI and is also one of the principal mechanisms of neurological injury. Consistent with the results of the PBS group, our previous research has demonstrated that neurons are regenerated 3–5 mm away from the injury site, while no neurons are regenerated at the injury site in the rat hit model^40^. Accordingly, the increased expression level of proteins associated with neuronal regeneration, i.e., NeuN, MAP2, and NF-200 in the Matrigel-alone group (although it was slightly improved) and the NSCs/Matrigel group indicates that Matrigel improves neuron regeneration at injury sites. Here, we must note that we did not differentiate whether the regenerating neurons were induced from endogenous NSCs or from undamaged neurons, nor did we detect the differentiation efficiency of the exogenous NSCs. As we intended to determine the possibility of using Matrigel as scaffold material for NSCs, we mainly investigated graft survival, neuronal regeneration in the graft, and behavioral recovery of the rat model. Further confirmation such as anterograde or retrograde tracing is necessary.

In the present study, the NSCs/Matrigel group had significantly improved hindlimb locomotion. Compared with the PBS group, the slightly improved BBB score in the Matrigel-alone group, while not statistically significant, is important for evaluating the effect of Matrigel on function. Behavioral recovery may be attributed to the mechanisms described above: better microenvironment provided by Matrigel for neuronal regeneration, reduced inflammatory response, lesion cavity filling, and cell replacement. In addition, factors such as cytokines and growth factors secreted by the transplanted NSCs also render it more conducive for regeneration^6^.

Collectively, our study demonstrates that, as a scaffold, Matrigel can support NSC survival and differentiation either *in vitro* or *in vivo*. The combination of Matrigel with NSCs can promote neuronal regeneration and functional recovery. Some previous studies on the combination of Matrigel with mesenchymal stem cells or embryo-derived NSCs in a dog model have shown results similar to ours. However, there are no studies clarifying the best combination. Cells co-cultured in Matrigel to treat a SCI model may achieve better improvement. Further study is necessary for this hypothesis. In addition, Matrigel, which is derived from mouse sarcoma, cannot be used clinically due to its potential immunogenicity and pathogen transmission. More efforts are needed to determine how it can be optimized so that it can be used clinically.

## Conclusions

Using biomaterials for stem cell transplantation for improved functional recovery after SCI is a promising prospect. We observed that the survival and differentiation of NSCs were improved after NSCs were mixed with Matrigel. After injection of NSCs combined with Matrigel into the injured spinal cord, regenerated neurons appeared in the injured spinal cord and the motor function of the SD rats were restored. These data suggest the therapeutic potential of Matrigel as a cell scaffold material for SCI repair.
